# Exploring the relationship between distress rumination, resilience, depression, and self-injurious behaviors among Chinese college athletes infected with COVID-19: a cross-sectional study

**DOI:** 10.3389/fpsyt.2023.1219867

**Published:** 2023-08-09

**Authors:** Xiuhan Zhao, Zongyu Liu, Liangyu Zhao, Liguo Zhang

**Affiliations:** School of Physical Education, Shandong University, Jinan, Shandong, China

**Keywords:** distress rumination, resilience, depression, mediation effect, self-injury, suicidality

## Abstract

**Objectives:**

Distress rumination is a cause of suicidality and self-injurious behavior (SSIB) among individuals. Although previous studies have shown that distress rumination, SSIB, resilience, and depression are significantly related, the interaction mechanism remains unclear. This study aimed to evaluate resilience and depression as mediators of the relationship between distress rumination and SSIB among Chinese college athletes infected with COVID-19.

**Methods:**

Convenience sampling was used to recruit participants from the National College Football Championship in Guangxi City, China from January to February 2023. Participants completed the Ruminative Responses Scale (RRS), a subscale of the Health-Risk Behavior Inventory (HBI), the Mental Toughness Index (MTI) and the Patient Health Questionnaire (PHQ-9). We used the PROCESS macro for SPSS to determine the mediating effect of resilience and depression between distress rumination and SSIB.

**Results:**

A total of 350 Chinese college athletes participated in this study and completed the questionnaire survey. 289 (81.7% boys; *M*_age_ = 20.31 years, *SD* = 1.60) of them have been infected with COVID-19. 59.9% (*n* = 173) participants were from urban areas and 15.6% (*n* = 45) of participants have engaged in self-injurious behaviors or suicidal ideation. College athletes’ distress rumination was significantly negatively correlated with resilience (*r* = − 0.28, *p* < 0.01), and was significantly positively correlated with depression (*r* = 0.49, *p* < 0.01) and SSIB (*r* = − 0.18, *p* < 0.01). Resilience was significantly negatively correlated with depression (*r* = − 0.35, *p* < 0.01) and SSIB (*r* = − 0.30, *p* < 0.01). Finally, depression was significantly positively correlated with SSIB (*r* = − 0.38, *p* < 0.01). Resilience and depression played a mediating role of the total effects of distress rumination and SSIB, respectively. Meanwhile, the chain mediating effect of resilience and depression was also significant.

**Conclusion:**

This study found that distress rumination can directly predict SSIB, and indirectly predict SSIB through the mediating effect of resilience and depression, and the chain mediating effect of resilience-depression. Therefore, reducing the degree of distress rumination of college athletes infected by COVID-19 and improving their resilience, as well as reducing their depression may help prevent SSIB.

## Introduction

Confinement as a result of the COVID-19 pandemic has led to changes in the lifestyle and mental health of athletes, which may expose them to certain psychological disorders (1). Taheri et al. (2) found that COVID-19 lockdowns had a negative impact on athletes’ mental health, leading to higher levels of depression, anxiety, and stress. As far as we know, little is known about the psychological impact of the COVID-19 pandemic lockdowns on college athletes, especially those infected by COVID-19. Therefore, it is important to study athlete behavior during the COVID-19 pandemic to identify factors and develop relevant intervention strategies that can help improve the consequences of COVID-19 pandemic or similar situations in the future. Suicidality is a complex series of processes (3), ranging from suicidal ideation, suicide planning, self-harm, suicide attempts, and ultimately death by suicide, which is rare before adolescence, but is very common among adolescents and college students worldwide. The prevalence of self-injurious behavior (e.g., cutting oneself, burning oneself) is also high among college students (4). Suicidality and self-injurious behavior are highly correlated with suicidal ideation and self-harm attempts (5), and are strongly associated with the finalization of suicidal behavior.

In current study, students who possess certain sporting abilities and pass the sports training examination of regular institutes of higher learning or the recruiting examination of elite sports teams are referred to as college athletes. In contrast to other college students, university athletes’ primary responsibility is to represent their institution by competing in local, state, and national competitions in order to support their institution’s and the country’s competitive sports programs. They are susceptible to mental health issues due to the overload of training, increased risk of injury, ambiguity around their sporting future, and ongoing pressure from school and competition (6). College athletes are the youth strength of the new era, with the dual pressure of education and training (7), and their suicidal behavior not only cause panic and emulation among their peers, but also damage the family structure and affect the safety building of schools and society. The stress-diathesis model (8) states that stressful events are an important factor in causing individuals to commit suicide. Stressful events, such as the outbreak of the COVID-19 pandemic, lead to the possibility that some college athletes may be infected by COVID-19 and develop psychological stress and distress regurgitation, which may culminate in a suicide behavior. Therefore, the issue of suicide among college athletes needs further attention, especially the study of SSIB among college students.

Distress rumination, a state in which individuals reflect on distressing events affected by negative life and emotions (9), is a potential factor that increases the risk of suicidal ideation (10). Studies have shown that college students who experience distressing ruminations after a traumatic event are more likely to have severe SSIB (11, 12). Similarly, another study found that distress rumination was a predictor of suicidal ideation (13). Furthermore, the stress-diathesis model shows that the behavioral and psychological disorders is the result of the interaction between stress and individual susceptibility (14). Whereas traumatic events are an important source of stress, painful rumination is a susceptibility factor that can jointly influence the level of suicidal ideation (15). It follows that distress rumination is an important predictor of suicidal ideation. Resilience is the ability of an individual to adapt well in the face of life adversity, trauma, tragedy, threat, or other major life stressors, and it implies the “ability to bounce back” in the face of life stresses and setbacks (16). Psychological resilience have been shown to have an impact on SSIB. Research has shown that mental resilience has a negative predictive effect on self-injurious behavior or suicidal ideation (17), and the lower the level of mental resilience, the higher the frequency of self-injury. Mental resilience not only serves as a protective factor to weaken the negative effects of negative events on individuals, but also reduces the likelihood of suicidal ideation (18).

Depression is a mood disorder characterized by depressed mood, including symptoms such as sadness and despair, reduced interest and inattention, which can jeopardize an individual’s academic life and interpersonal interactions, and even lead to suicide in severe cases (19). Numerous studies have shown that depression is an important risk factor for triggering SSIB and is strongly associated with suicidal ideation and behavior (20–22). Suicide is the most serious consequence of depression, where the prevalence of depression is about 2 to 9% (23). A Meta-analysis showed that the type of mental disorder most strongly associated with suicide attempts was depressive disorder (24). According to Eriksons theory of psychological development, college students are at a critical stage of rapid physical and mental development and are vulnerable to psychosocial factors (25). Seo et al. (26) have found that reducing the level of depression in individuals plays an important role in suppressing suicidal ideation and reducing suicidal behavior. Besides, the interaction between stressful life events and ruminative thinking has been found to increase the risk of depressive symptoms (27). Research has found that distress rumination, as a negative cognitive style, is a key susceptibility factor for depression (28). And individuals with higher levels of ruminative thinking experience more intense negative emotions, which subsequently increase the risk of developing depressive mood.

However, there are few studies on college athletes that support this pathway. Therefore, the main purpose of this study was to explore the relationship and mechanisms of distress rumination, resilience, depression, and SSIB in our college athletes, which can play an important role in reducing SSIB and improving quality of life in college athletes. Four hypotheses were proposed in this study: (H1) distress rumination is related to SSIB; (H2) resilience may play a mediating role between distress rumination and SSIB; (H3) depression may play a mediating role in distress rumination and SSIB; (H4) resilience and depression may play a mediating role in distress rumination and SSIB (H3) depression may play a mediating role in distress rumination and SSIB; (H4) resilience and depression may play a chain mediating role in distress rumination and SSIB.

## Methods

### Participants

This is a cross-sectional study. Participants were recruited from a National College Football Championship in Guangxi City from January to February 2023. The COVID-19 pandemic has been going on for nearly 3 years, and there is still no end in sight (29). During this phase, many athletes were infected by COVID-19 due to the widespread spread of the COVID-19 pandemic in China. Convenience sampling method was used in this study. The college athletes included in this study had some level of athletic certification, were ≥ 18 years old, and provided written informed consent.

### Data collection

Participants were recruited after approval by the Ethics Committee of Shandong University, China (No. 2021–1-114). The researcher (Liguo Zhang) were uniformly trained in the data collection methods before the formal investigation. The included sampling criteria are Chinese university athletes who are willing to participate in the research, have participated in national competitions and have the grade certificate of Chinese athletes. Exclusion criteria were (1) unfinished questionnaires, (2) those with severe physical and mental illnesses, or (3) the inability to provide written informed consent. Data were gathered through in-person interviews. The eligible participants were first told of the study’s objectives, and those who agreed to participate completed an informed consent form before the formal inquiry could begin. The participants answered the questionnaire on their own after the researchers had presented the standardized instructions for the data collection tool. The researchers were on hand to answer any questions or handle any issues that the participants had. The researcher read each questionnaire item and recorded participants’ responses if they were unable to finish it on their own. The questionnaire took between 15 and 20 min to complete. To prevent the need for a second examination, completed questionnaires were immediately reviewed for errors.

### Measures

#### Demographic characteristics

Sociodemographic questions in the questionnaire gathered pertinent data on participant characteristics such gender, age, urban/rural provenance, athletic level, and daily screen entertainment time.

#### Distress rumination

The Chinese version of the Ruminative Responses Scale (RRS) was used to assess distress rumination after infected by COVID-19 (30). The RRS is a valid and reliable tool that consists of 22 self-reported items, each rated on a scale of 1–4. The items are summed to create a total score that reflects the tendency to ruminate when dealing with negative information (total score: 22–88). The reliability and validity of the Chinese version of instrument have been shown by a previous study (31). The Cronbach’s α coefficient of the scale in this study was 0.966.

#### SSIB

The Chinese version of the 5-item questionnaire, a subscale of the Health-Risk Behavior Inventory (HBI), was used to assess SSIB (32). Specifically, the following two items evaluated self-injurious behavior of college athletes who were infected with COVID-19: (a) Have you ever intentionally cut or burned yourself after contracting a new crown and (b) Have you ever thought about biting, scratching or hitting yourself? The other three items examined suicidality as follows: (a) Have you ever seriously thought about killing yourself? (suicide ideation), (b) Have you ever had a plan on how to end your life? (suicide plan), (c) Have you ever tried to commit suicide? (suicide attempt). Respondents reported the frequency in which they engaged in each behavior after infected by COVID-19 from 0 (never) to 4 (always). The HBI has a strong test–retest reliabilities and external validities, which can distinguish high risky respondents effectively (33). The Cronbach’s α coefficient of this subscale in this study was 0.960.

#### Resilience

The Chinese version of the Mental Toughness Index (MTI) was used to assess resilience (34). The scale consists of 8 items that measure mental toughness. Items (e.g., “I try to continue to be successful in tennis”) are rated on a 7-point response scale from 1 (incorrect, 100% of the time) to 7 (correct, 100% of the time). The reliability and validity of the instrument have been shown by previous studies in a Chinese athletes sample (35). The Cronbach’s α coefficient of the scale in this study was 0.972.

#### Depression

The Chinese version of the Patient Health Questionnaire (PHQ-9) was used to assess depression (36). The questionnaire consists of 9 items to assess depressive symptoms, each item is scored as not at all = 0, a few days = 1, more than half the time = 2, and almost every day = 3. The score is moderate depression, 15–19 is moderate to severe depression, and 20–27 is severe depression. The reliability and validity of the Chinese version also has been supported in Chinese college samples (37). The Cronbach’s α coefficient of the scale in this study was 0.923.

### Statistical analysis

Data analysis was done using SPSS version 22.0. While frequency is used to represent categorical data, mean and standard deviation are used to explain continuous variables. To examine the association between the variables, we employed Pearson’s bivariate correlation analysis. Hayes employed Model 6 in the PROCESS macro to evaluate the chain mediation effect (38). When estimating the chain mediation effect from 5,000 samples, the regression coefficients were tested for significance using the Bootstrap 95% confidence interval. The indirect effect was deemed statistically significant if the 95% confidence interval (CI) did not include 0. Additionally, we employed an structural equation model (SEM) made by AMOS 22.0 to validate the proposed model. In this study, model fit was assessed using descriptive fit indices. The CFI, TLI, RMSEA, and SRMR are the descriptive fit indices that have been most frequently examined as confirmatory factor analyses and structural equation modeling indicators (39). In the current study, we adopted the methodology of Accurso et al. (39), which represents a good fit (or well-fitting) model by CFIs and TLIs≥0.95 (0.90 – 0.94), RMSEA <0.05 (to 0.08), and SRMR <0.05 (to 0.08) (40). The model is regarded as having a good fit if three out of the four descriptive markers show this.

## Results

### Common method deviation test

All variables in this study were included in exploratory factor analysis using Harman’s single factor test. The results showed there were 7 factors with eigenvalues >1. The first factor explained 34.76% of the total variance, which was lower than the critical value of 40%. Therefore, there was no serious common method deviation in the data used in this study.

### Sociodemographic characteristics

A total of 350 Chinese college athletes participated in this study and completed the questionnaire survey. After data screening, 289 of them have been infected by COVID-19. Therefore, a total of 289 Chinese athletes infected by COVID-19 participated in this study and completed the questionnaire survey. Participants’ average age was 20.31 (standard deviation was 1.60) years old (range 18–28 years); 15.6% (n = 45) of participants have engaged in self-injurious behaviors or suicidal ideation. Table 1 presents participants’ sociodemographic characteristics.

**Table 1:** Socio-demographic characteristics of participants.

Variables	Socio-demographic characteristics	Participants (*N* = 289)
Gender, *N* (%)	Male	236 (81.7)
	Female	53 (18.3)
Age, Mean (SD)		20.31 (1.60)
Urban–rural provenance, *N* (%)	Urban	173 (59.9)
	Rural	116 (40.1)
Duration of sports careers, *N* (%)	1–5 years	63 (21.8)
	6–10 years	153 (52.9)
	11–13 years	51 (17.6)
	14 years or more	22 (7.6)
Athlete level, *N* (%)	Second grade	184 (63.7)
	First grade	100 (34.6)
	National grade	5 (1.7)
Daily screen entertainment time, *N* (%)	None	3 (1)
	Less than 2 h	55 (19)
	2–3 h	111 (38.4)
	3–5 h	85 (29.4)
	Over than 5 h	35 (12.1)

### Descriptive data of participants’ distress rumination, resilience, depression and SSIB divided by gender

The mean (standard deviation) of distress rumination, resilience, depression and SSIB was 14.89 (12.95), 67.77 (20.92), 2.90 (4.63), and 6.13 (3.33), respectively (Table 2). The results showed that only resilience was significantly different between groups.

**Table 2:** Descriptive data of participants’ distress rumination, resilience, depression and SSIB divided by gender.

	Total Mean ± SD	Males’ Mean ± SD	Females’ Mean ± SD	*T*	*p*-Value
Distress rumination	14.89 ± 12.95	15.02 ± 12.57	14.30 ± 14.64	0.363	0.743
Resilience	67.77 ± 20.92	68.87 ± 21.20	62.87 ± 19.08	1.897	0.046^*^
Depression	2.90 ± 4.63	2.77 ± 4.52	3.49 ± 5.09	−1.023	0.346
SSIB	6.13 ± 3.33	6.15 ± 3.41	6.06 ± 2.99	0.189	0.838

*p < 0.05.

### Correlation analysis of distress rumination, resilience, depression and SSIB in Chinese college athletes who were infected with COVID-19

Correlation analysis of all variables showed that distress rumination was significantly negatively correlated with resilience (*r* = − 0.28, *p* < 0.01), and was significantly positively correlated with depression (*r* = 0.49, *p* < 0.01) and SSIB (*r* = − 0.18, *p* < 0.01). Resilience was significantly negatively correlated with depression (*r* = − 0.35, *p* < 0.01) and SSIB (*r* = − 0.30, *p* < 0.01). Finally, depression was significantly positively correlated with SSIB (*r* = − 0.38, *p* < 0.01) (Table 3).

**Table 3:** Statistical description and related analysis results.

	M	SD	1	2	3	4
1	14.89	12.95	1.00			
2	67.77	20.92	−0.28^**^	1.00		
3	2.90	4.63	0.49^**^	−0.35^**^	1.00	
4	6.13	3.33	0.18^**^	−0.30^**^	0.38^**^	1.00

1 = distress rumination; 2 = resilience; 3 = depression; 4 = SSIB. **p < 0.01.

### Test of the mediating effect of resilience and depression in Chinese college athletes who were infected with COVID-19

Model 6 in the PROCESS macro compiled by Hayes was used to test the mediating effect of resilience and depression between distress rumination and SSIB. The structural equation model was used to verify the model, and the fitting index was: χ^2^/df = 2.1. CFI = 0.98; TLI = 0.94; SRMR = 0.03; RMSEA = 0.06, indicating that the model has an acceptable fitting effect. The regression results (Table 4) showed that the distress rumination for Chinese college athletes infected by COVID-19 was not directly correlated with SSIB (β = − 0.03, *p* > 0.05). The distress rumination negatively predicted resilience (β = − 0.29, *p* < 0.01) and positively predicted depression (β = 0.42, *p* < 0.05). Resilience negatively predicted depression (β = − 0.23, *p* < 0.01) and SSIB (β = − 0.20, *p* < 0.01). Finally, depression positively predicted SSIB (β = 0.33, *p* < 0.01).

**Table 4:** Regression analysis among variables in the chain intermediary model.

Outcome variable	Predictor variable	*R*	*R* ^2^	*F*	*β*	*t*
Resilience	Distress rumination	0.32	0.10	4.46^**^	−0.29	−4.99^**^
Depression	Distress rumination	0.55	0.30	14.83^**^	0.42	7.75^**^
	Resilience				−0.23	−4.40^**^
SSIB	Distress rumination	0.44	0.19	7.34^**^	−0.03	−0.50
	Resilience				−0.20	−3.45^**^
	Depression				0.33	5.17^**^

^**^p < 0.01. The demographic variables are controlled as covariates.

**Figure 1:**
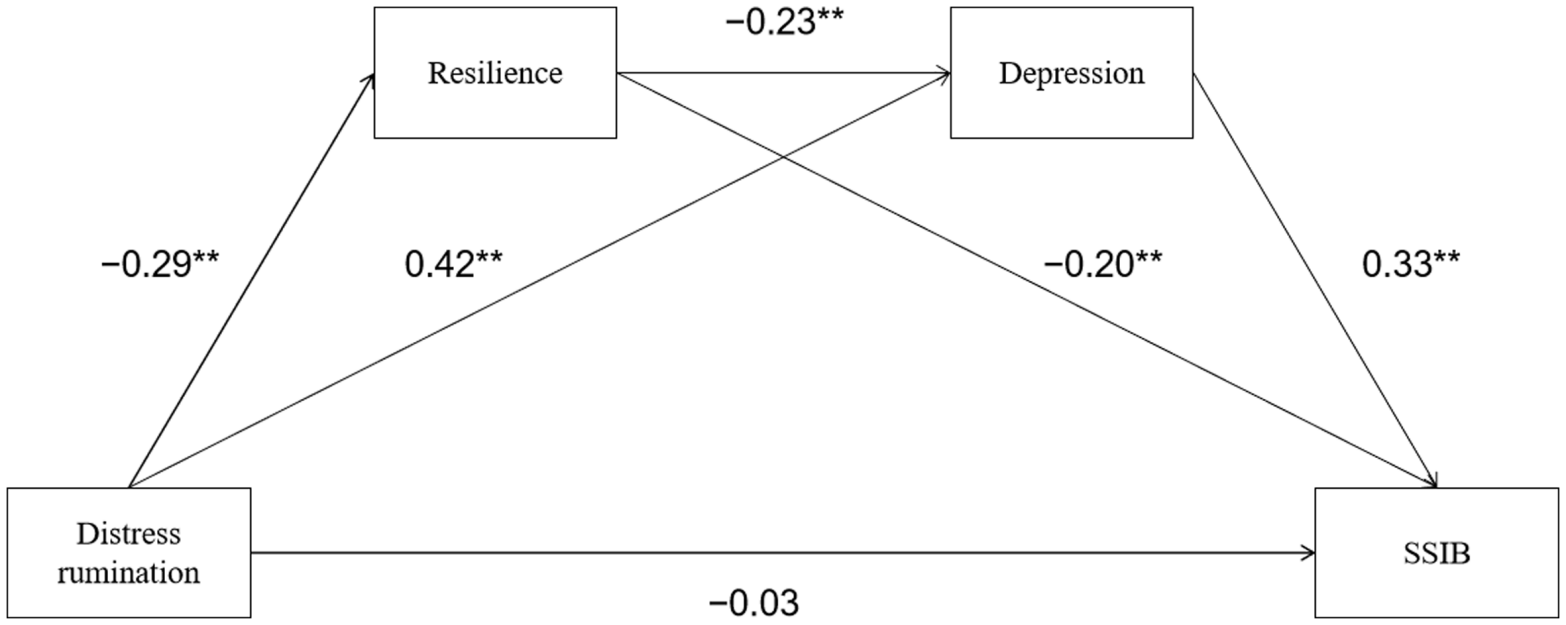
The chain mediating effect of resilience and depression on the chain between distress rumination and SSIB. ^**^p < 0.01.

To further examine the mediation effect, the non-parametric percentile bootstrap technique of deviation calibration was applied. The findings demonstrated that the 95% confidence interval (CI) for each path did not include 0, demonstrating that the mediation effect was significant and establishing chain mediation (Table 5). The specific path of the relationship between distress rumination and SSIB in Chinese college athletes who were infected by COVID-19 is shown in Figure 1. The direct effect of distress rumination on SSIB is not significant, suggesting both resilience and depression play a complete mediation effect in the relationship between distress rumination and SSIB.

**Table 5:** Analysis of the mediating effect of resilience and depression.

	Effect size	Standard error	Boot CI LL	Boot CI UL	Relative mediation effect %
Direct effect	−0.03	0.06	−0.16	0.09	0%
Indirect effect 1	0.06	0.07	0.01	0.12	27.27%
Indirect effect 2	0.14	0.05	0.03	0.23	63.64%
Indirect effect 3	0.02	0.02	0.01	0.06	9.09%
Total mediation effect	0.22	0.07	0.08	0.34	100%

Indirect effect 1: Distress rumination → Resilience → SSIB. Indirect effect 2: Distress rumination→Depression→SSIB. Indirect effect 3: Distress rumination→ Resilience→Depression→SSIB. The demographic variables are controlled as covariates.

## Discussion

SSIB among athletes is worthy of attention among college athletes (41), especially during and after the COVID-19 pandemic. Our major findings were as follows: first, distress rumination was associated with SSIB in college athletes who were infected with COVID-19; second, depression mediated the association between distress rumination and SSIB; third, resilience mediated the association between distress rumination and SSIB; fourth, depression and resilience played a serial mediating role between distress rumination and SSIB. In general, the results supported our four hypotheses.

This research found that resilience played a part of the mediating role between distress rumination and SSIB in Chinese college athletes who were infected with COVID-19, and the mediating effect accounted for 27.27% of the total effect. A previous study showed that distress rumination among college students was negatively correlated with resilience (42); that is, the more severe the distress rumination, the lower the level of resilience. The same result was obtained in this study. Individuals who ruminate on their distress may not be able to concentrate on their task or training because of the painful thoughts, resulting in a range of negative emotions and negative perceptions about the future (9), thus reducing their level of resilience. Resilience may reduce the negative impact of distress rumination on SSIB. Mental toughness, as an important indicator to resist external stress, can give timely defusion and recovery to maintain a good mental state when encountering setbacks (43). Research has shown that psychological resilience can improve individuals’ negative emotions in the face of adversity and can serve as an internal defense mechanism to regulate SSIB (44). And high-level resilience increases the resistance to negative emotions precisely when college athletes infected with COVID-19 engage in distress rumination, allowing them to confront this public health emergency. As a result, they will be able to actively mobilize their positive energy against frustration and reduce distress rumination (16, 18). Targeted individualized health education can be provided during the care process to allow them to fully understand the process of recovery from infection and cooperate with treatment; at the same time, modern communication media can be used to establish social groups to share past recovery experiences, build confidence and increase psychological resilience from both the medical and nursing perspectives. In addition, transfer stimulation can be used to mitigate their falling into negative thinking when they are alone by using music, radio, and meditation training appropriately, thus reducing the degree of influence of ruminative thinking on SSIB. Besides, from the perspective of school education, sports teams can be equipped with special psychological intervention counselors.

The results of this study showed that depression played a partial mediating role between distress rumination and SSIB, which was consistent with the previous research result (45). The mediating effect of depression accounted for 63.64% of the total effect, indicating that depression plays the most important mediating role in the present study. Depression, as a negative emotional experience can lead to a series of cognitive-behavioral impairments in severe cases, increasing the individual’s intrinsic harmfulness, such as suicidal ideation and suicidal behavior (46). Depressed individuals are chronically immersed in negative emotions, their social functioning is impaired, their sense of belonging in interpersonal interactions is low and their sense of burden is high, and individuals can develop suicidal ideation (47). Zhang et al. (48) showed that the level of distress rumination can predict the depression of adolescents, which was consistent with the conclusions of this study. A stressful event (such as infected by COVID-19) can disrupt an individual’s ability to self-regulate and solve problems, reduce the utilization of social support, adversely affect the utilization of superior resources in college athletes, and aggravate the level of painful rumination. As a typical response style, distress rumination is an important cognitive risk factor for depression (49). Hyperrumination is characterized by excessive attention to negative information, difficulty in controlling or stopping rumination, and can also associate the self with negative information, thus perpetuating negative cognitive tendencies, which in turn cause and sustain depression. When college athletes who were infected with COVID-19 have high-level distress rumination, they will repeatedly think about the dangers of COVID-19 pandemic as well as their own negative emotions and behaviors, exacerbating depressive symptoms. Additionally, depressed college athletes who have a high propensity to ruminate raises their sense of hopelessness, which is a significant risk factor for the emergence of suicidal ideation and eventually raises the probability of suicidal behavior (25, 28). Therefore, depression plays a mediating role in the prediction effect of rumination on SSIB of college students. For government and schools, it is important to reduce the depression of college athletes who were infected with COVID-19 to reduce the impact of distress rumination on SSIB.

Finally, the distress rumination level of college athletes who were infected by COVID-19 can also affect SSIB through the chain mediating effect of resilience and depression, and the mediating effect accounted for 9.09% of the total effect. The results of this study showed that resilience was negatively correlated with depression, indicating that the higher an individual’s resilience, the lower their level of depression. It is found that resilience, as an internal positive coping mechanism for individuals to cope with negative life events, can cushion depression. College athletes with higher mental toughness level in orthopedics have stronger confidence and courage in the face of difficulties, which has a positive impact on the improvement of their depressive symptoms. Study have shown that individual resilience has a significant negative predictive effect on depression (50), and resilience plays a certain role in the relationship between negative life events and depression. That is, high individual resilience can alleviate the negative impact of negative life events. According to Lazarus’ stress coping theory (51), individuals will spontaneously cope with stressful events through a series of emotions (such as depression) or behavioral strategies. Meanwhile, the resources available to them (such as resilience) will in turn regulate their efforts. Based on this, we speculate that the level of resilience of college athletes who were infected by COVID-19 might be reduced if they constantly regurgitated the pain caused by the COVID-19 event. After the level of mental resilience is reduced, they will have a harder time regulating their emotions, so their depression level may rise, the inner negative emotions are difficult to resolve, and eventually lead to SSIB. According to the mixed model of emotional intelligence (52), emotional intelligence is a mental health protective factor that includes five key components: self-awareness, self-management, internal drive, empathy, and social skills, and has been shown to be significantly associated with depression and resilience. Individuals with distress rumination are generally immersed in a state of negative emotions, exhibit weaker self-management skills, have difficulty regulating their emotions, and eventually tend to develop suicidal ideation or self-injurious behavior.

In contrast to the mediation effect, which can clearly analyze both direct and indirect effects between variables and explain the internal relationships, prior studies have mainly concentrated on the application of multiple logistic regression to explore the influencing factors of SSIB. The internal mechanism of distress rumination on SSIB is clarified by our research findings. To the best of our knowledge, our research is the first to discover that there is a chain mediating effect of resilience and depression in the effect of distress rumination on SSIB. That is to say, our research hypothesis is valid, which provide theoretical basis and empirical support for reducing or delaying the occurrence of SSIB among college athletes who were affected with COVID-19. Practically, the likelihood of development to SSIB may be decreased by identifying distress rumination and intervening with their depression and resilience to meet their requirements. College athletes who were infected with COVID-19 can be guided and assisted by the government or educators to lessen their distress rumination (e.g., by providing timely psychological counseling), in the true sense of encouraging and creating conditions for them to lessen depression, and enhance their resilience, thereby reducing the likelihood of their SSIB. For college athletes who frequently engage in distress rumination, positive individualized mental health interventions can be implemented in schools to help them better manage the dual stresses of coursework and training, reduce their SSIB, improve their well-being, and ultimately enhance their competition performance.

There were some limitations in our study. First, the design of the cross-sectional study reduced the ability to draw causal correlations between variables. Second, this study’s conclusions may not apply to collegiate players from different backgrounds. In addition, the sample selected for this study was soccer athletes and it is worth further testing whether this mechanism still exists in other types of athletes (e.g., badminton players, basketball players).

## Data availability statement

The raw data supporting the conclusions of this article will be made available by the authors, without undue reservation.

## Ethics statement

The studies involving human participants were reviewed and approved by Ethics approval was obtained from the Ethics Committee of Shandong University (No. 2021-1-114). All methods comply with the current guidelines and regulations which follow the Declaration of Helsinki. The participants provided their written informed consent to participate in this study. Written informed consent was obtained from the individual(s) for the publication of any potentially identifiable images or data included in this article. The patients/participants provided their written informed consent to participate in this study.

## Author contributions

This study was designed and carried out by LigZ and XZ, who also gathered data. LiaZ organized and evaluated the data. The first draft was written by XZ and ZL. LiaZ and LigZ took part in the project’s statistical analysis phase. LigZ offered thorough feedback on various sections of the paper. All authors contributed to the article and approved the submitted version.

## Funding

This research was supported by the Social and Science Planning Project of Shandong Province (no. 20DTYJ03) and the Graduate Research Innovation Fund of Shandong University (no. 2021YJKTYB06).

## Conflict of interest

The authors declare that the research was conducted in the absence of any commercial or financial relationships that could be construed as a potential conflict of interest.

## Publisher’s note

All claims expressed in this article are solely those of the authors and do not necessarily represent those of their affiliated organizations, or those of the publisher, the editors and the reviewers. Any product that may be evaluated in this article, or claim that may be made by its manufacturer, is not guaranteed or endorsed by the publisher.
